# Post-Operative Delirium and Cognitive Decline in Kidney Transplant Recipients

**DOI:** 10.1093/geroni/igaa057.1677

**Published:** 2020-12-16

**Authors:** Nadia Chu, Xiaomeng Chen, Dorry Segev, Mara McAdams-DeMarco

**Affiliations:** 1 Johns Hopkins School of Medicine, Bethesda, Maryland, United States; 2 Johns Hopkins School of Medicine, Baltimore, Maryland, United States; 3 Johns Hopkins Bloomberg School of Public Health, Baltimore, Maryland, United States

## Abstract

Post-operative delirium may be a marker for cognitive reserve, or a greater cognitive vulnerability to stressors. As a result, those with post-operative delirium may experience steeper decline in cognitive performance following stressors of surgery post-KT. We leveraged a single-center cohort study of 912 adult KT recipients with delirium assessments abstracted from medical records. Global cognitive function (3MS) and executive function (time to complete TMT-B minus TMT-A) were measured at time of KT, 1-month, 3-months, 6-months, 1-year, and annually thereafter post-KT. We used mixed effects models with fixed and random effects for person and time to describe repeated measures of cognitive performance and compared trajectories by post-operative delirium. Among 912 KT recipients, 44 (4.8%) had post-operative delirium. Post-operative delirium was associated with higher levels of cognitive impairment at KT (18.2% vs 8.0%), and was associated with lower 3MS component scores including in memory, identification/association, and orientation. After adjustment, those with delirium had 3MS scores that were on average 3.6 points lower than those without delirium (95%CI: -6.9, 0.3) at time of KT; delirium was not associated with differing global cognitive trajectories post-KT (difference=0.04 points/month, 95%CI:-0.1, 0.2). However, delirium was associated with lower executive function at KT (difference=44.0s, 95%CI: 17.4, 70.6) and steeper decline in executive function post-KT (difference=-1.1s/month, 95%CI:-2.1,-0.05). KT recipients with delirium experience greater decline in executive function, indicating greater cognitive vulnerability with potential vascular etiologies. Transplant centers should be aware of the cognitive risks associated with post-KT delirium and implement available preventative interventions to reduce risk of delirium.

